# Self-Shielding Gyroscopic Radiosurgery for Uveal Melanoma: A First Case Report

**DOI:** 10.7759/cureus.59859

**Published:** 2024-05-08

**Authors:** Paul Foerster, Julian Klaas, Christoph Furweger, Felix Ehret, Alexander Muacevic, Antonio Santacroce

**Affiliations:** 1 Department of Ophthalmology, University Hospital of Munich Ludwig Maximilian (LMU), Munich, DEU; 2 Department of Radiosurgery, European Radiosurgery Center Munich, Munich, DEU; 3 Center for Neurosurgery, Department of Stereotaxy and Functional Neurosurgery, University of Cologne, Cologne, DEU; 4 Department of Radiation Oncology, German Cancer Consortium, German Cancer Research Center, Charité – Universitätsmedizin Berlin, Berlin, DEU; 5 Department of Medicine, Faculty of Health, Witten/Herdecke University, Witten, DEU; 6 Department of Neurosurgery, St. Barbara-Klinik Hamm-Heessen, Hamm, DEU

**Keywords:** choroidal malignant melanoma, zap gyroscopic radiosurgery, eye cancer, single fraction stereotactic radiosurgery, uveal melanoma

## Abstract

Stereotactic radiosurgery (SRS) is a well-established treatment modality for the management of uveal melanoma, achieving high tumor control and eye retention rates. There are several SRS treatment platforms available, including the recently developed self-shielding gyroscopic radiosurgery (GRS) system. We report the first use of GRS in the treatment of uveal melanoma.

We report the treatment of a 63-year-old female patient with a left-sided uveal melanoma. Akinesia of the ocular globe in the orbit was achieved by retrobulbar anesthesia. The treatment plan used six isocenters (three with the 10 mm and three with the 7.5 mm apertures) and 140 beams to cover 99.2% of the planning target volume (PTV) with 21 Gy at the 54% isodose line. Treatment was delivered in a single session with the GRS device. The total workflow time from retrobulbar anesthesia to completion of treatment was 122 minutes.

The procedure was flawless, clinically well tolerated by the patient, and reliably performed in an outpatient setting, thus comparable to our published experience with robotic SRS. The evaluation of new radiosurgery treatment platforms is critical to maintaining quality standards and refining future treatments.

## Introduction

Although rare, uveal melanoma is the most common primary intraocular malignancy in adults, with an incidence of six to seven per million and bears a dismal prognosis when metastatic [1]. As uveal melanoma is rarely symptomatic in its early stages, it is typically diagnosed either during routine check-ups or in later stages, when a reduction in visual acuity occurs. It is notable that there is no influence of gender in the development of uveal melanoma. This condition is significantly more common in Caucasians than in other ethnicities, with a peak incidence occurring at around 60 years of age. The Collaborative Ocular Melanoma Study (COMS) has shown that there is no significant difference in metastasis and overall survival rates between patients treated with enucleation and those treated with brachytherapy. Therefore, eye preservation is possible and desirable [1]. Furthermore, radiation therapy achieves high tumor control, thus avoiding enucleation of the ocular globe [2-4]. Brachytherapy with radioactive ocular plaques is limited to patients with uveal melanomas of limited height and transverse diameter. However, teletherapy, such as fractionated proton beam therapy, conventional linear particle accelerator (LINAC) radiotherapy, and frame-based radiosurgical techniques, can be successfully applied to larger tumor volumes, as well as tumors located close to the posterior pole of the eye orbit. Each modality of radiotherapy has its own advantages and risks of radiation-related complications. In recent years, single-fraction frameless robotic radiosurgery has proven to be a safe and effective treatment modality for patients with uveal melanoma. Recently, the first self-shielding radiosurgery platform that allows frameless gyroscopic radiosurgery (GRS) has been introduced [5]. GRS provides the possibility to conduct radiosurgical treatments without the need for radiation vaults, potentially increasing the availability of this treatment modality. This is the first report of a patient harboring a uveal melanoma treated with GRS. This case report aims to describe the safety and feasibility of the procedure and our initial institutional treatment experience.

## Case presentation

**Figure 1:**
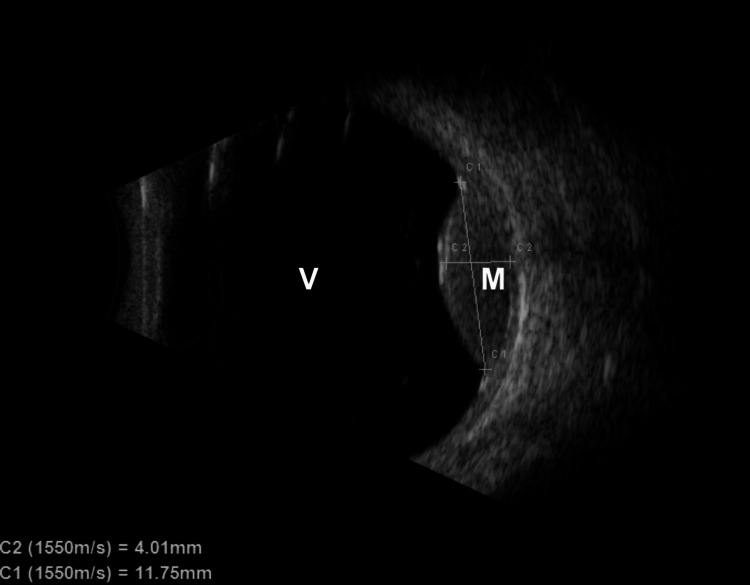
Ultrasound eye examination before treatment. V: vitreous, M: melanoma.

**Figure 2:**
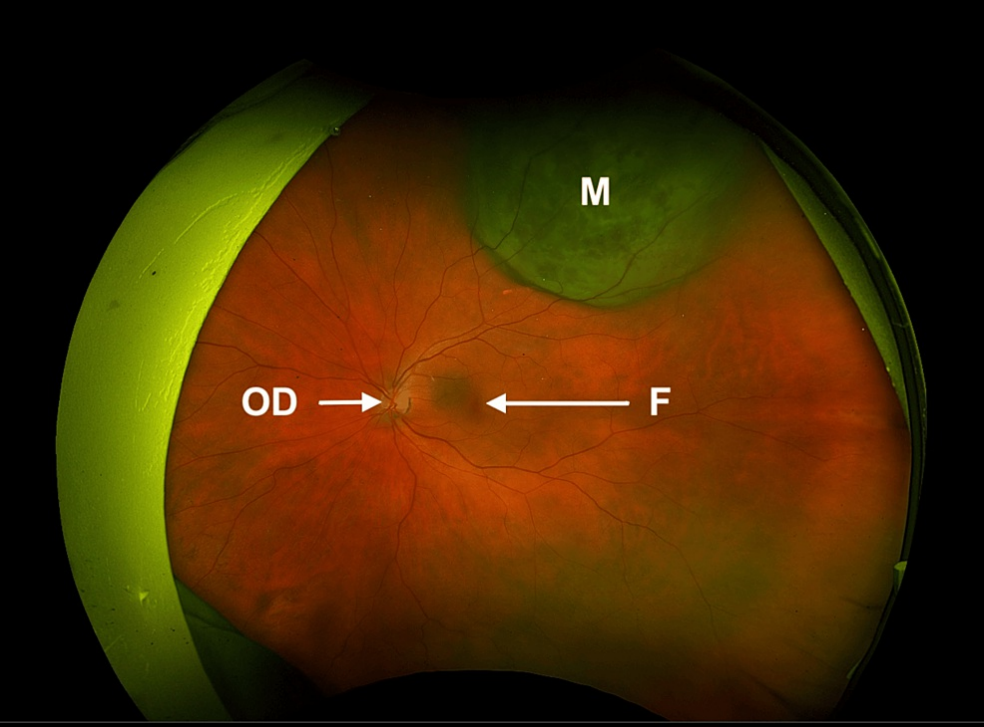
Fundus: retinal wide-field imaging of the left eye before treatment. OD: optic disc, F: fovea, M: melanoma.

Upon initial presentation, the 63-year-old female patient received a complete ophthalmologic examination with an assessment of visual acuity, indirect ophthalmoscopy, and standardized A-/B-scan ultrasound (ABSolu Quantel Medical, France) (Figures 1, 2). Written consent for treatment was obtained after an explanation of the benefits and risks of the various treatment options, and the patient was subsequently enrolled in the prospective study with a Clinical Trials Identifier: DRKS00025820.

GRS was performed on January 17th, 2024 as an outpatient procedure by adapting a well-established workflow for robotic radiosurgery [3]. First, akinesia of the globe within the orbit was achieved by retrobulbar anesthesia. Magnetic resonance images (MRI, 3T Magnetom Vida, Siemens, Germany, T2, and gadolinium-contrast enhanced T1) of the orbit were acquired with 1.0- and 1.1-mm axial slices. Subsequently, the patient was positioned on the couch of a computed tomography (CT) (Somatom Definition AS, Siemens, Germany) scanner. A three-point thermoplastic mask (Orfit Industries, Wijnegem, Belgium) was molded to establish a reproducible head position and patient immobilization. A planning CT scan of the patient was acquired with 1.0 mm axial slices. CT and MRI scans were overlaid for contouring of the target and organs at risk, such as the optic disc, fovea, lens, and contralateral eye. A planning target volume (PTV) was created by adding a 1-mm isotropic margin and an additional 1 mm posteriorly to the visible tumor. The posterior expansion was intended to compensate for a slight posterior shift of the eyeball caused by anesthetic resorption during treatment. 

A GRS treatment planning system (v1.8.59, ZAP Surgical Inc., San Carlos, CA, USA) was utilized to place isocenter coordinates inside the PTV. A total of 459 non-coplanar candidate beams of different diameters were targeted at the isocenter coordinates. The contralateral eye was blocked for beam traversal. The weights of candidate beams were optimized with an inverse algorithm to achieve a dose distribution conformal to the PTV and a steep falloff towards the surrounding healthy tissue. Beams of low weight were discarded. A standard dose of 21 Gy was prescribed for the 54% isodose encompassing the PTV. For comparison with an established standard, a treatment plan for robotic radiosurgery was created (Precision v3.4 for the CyberKnife, Accuray Inc., Sunnyvale, USA). 

Treatment was delivered in a single session with a ZAP-X system (ZAP Surgical Inc., San Carlos, CA, USA), which uses a compact 3 MV LINAC, a collimator wheel with eight circular apertures, and kV image guidance [5,6]. Coupled gimbals are employed to position the LINAC for non-coplanar beam delivery. Intrafraction of patient motion was compensated by acquiring kV images every 40 seconds, deriving the offset of the patient's head by comparison with digitally reconstructed radiographs and automatically moving the couch. After completion of the treatment, the mobility of the globe was assessed by ophthalmologic examination.

**Figure 3:**
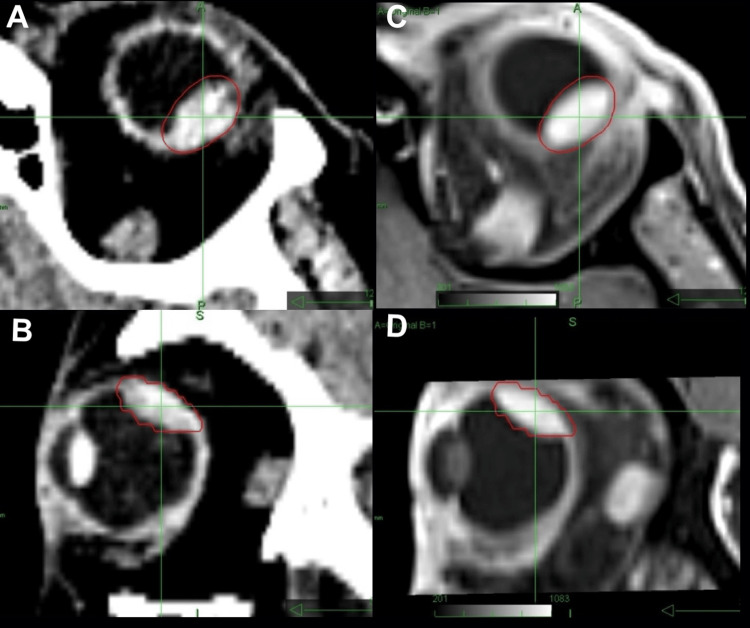
CTV: Tumor volume (red) on CT axial (A) and sagittal (B) and contrast-enhanced T1 MRI in axial view (C) and sagittal view (D). CTV: clinical target volume, MRI: magnetic resonance image.

The best-corrected visual acuity was 0.8 for both eyes. Ultrasound examination revealed a left-sided peripheral uveal melanoma with base dimensions of 11.8 mm by 11.3 mm and a height of 4.3 mm (Figure 1). The volume of the visible tumor was 0.53 cm³ in CT and MR imaging (Figure 3), which resulted in a planning target volume (PTV) of 0.97 cm³. Table 1 summarizes plan quality metrics and doses to the target and at-risk organs in comparison to the robotic radiosurgery treatment plan.

**Table 1:** Treatment plan characteristics. Data for the reported patient has been represented as gray (Gy), percent (%), volume (cc), number (#), and time (min). SRS: stereotactic radiosurgery, GRS: gyroscopic radiosurgery.

	Metric	GRS	Robotic SRS
Prescription	Dose (Gy)	21	21
	Isodose (%)	54	70
PTV	New conformity index	1.25	1.16
	Homogeneity index	1.85	1.43
	Coverage (%)	99.2	99.3
	Mean dose (Gy)	28.2	25.7
Global	Gradient index	2.71	3.24
	V10Gy (cc)	3.42	3.84
Lens	Max dose (Gy)	5.2	5.4
	Mean dose (Gy)	2.9	2.9
Optic disc	Max dose (Gy)	4.1	3.0
	Mean dose (Gy)	3.7	2.6
Fovea	Max dose (Gy)	4.6	4.4
	Mean dose (Gy)	4.5	4.0
Delivery	Beams (#)	140	97
	Monitor units (#)	8520	6290
	Time est. (min)	34	26

**Figure 4:**
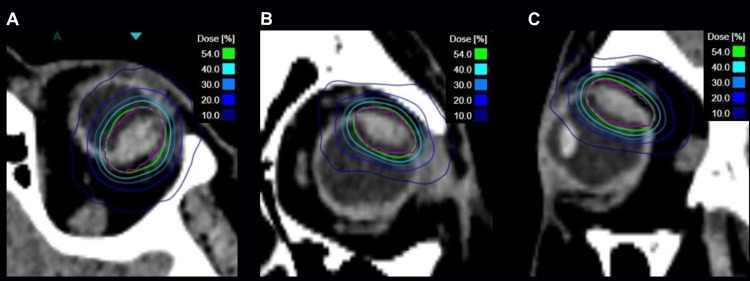
Isodose distribution around the PTV (purple) in axial (A), coronal (B), and sagittal (C) views. The green 54% isodose line represents the prescription dose of 21 Gy. PTV: planning target volume.

The final plan used six isocenters (three with the 10 mm and three with the 7.5 mm apertures) and 140 beams to cover 99.2% of the PTV with 21 Gy at the 54% isodose line (Figure 4). 

Overall, the duration of the workflow from retrobulbar anesthesia until completion of the treatment was 122 minutes, with 34 minutes attributed to MRI and CT imaging, 26 minutes for image fusion and contouring, 11 minutes for plan optimization, 16 minutes for patient preparation and setup, and 35 minutes for treatment delivery (Table 2).

**Table 2:** Timeline for the entire treatment.

	Time
Start time	9:10
Retrobulbar anesthesia achieved	9:22
MRI completed	9:50
CT completed	9:56
Image data fused in treatment planning system (TPS)	10:05
Target and organs at risk (OAR) contoured	10:22
Plan optimized and approved	10:33
Setup completed	10:49
Treatment completed	11:24

Akinesia of the globe persisted until the end of the treatment. The patient was discharged home after the end of the procedure. Follow-up is planned at three, six, 12, and 18 months and yearly, thereafter with clinical, ultrasound, and MRI studies. Seven days after radiosurgery, the patient did not experience any treatment-related side effects.

## Discussion

The goal of radiotherapy in treating uveal melanoma is to achieve tumor control and ocular preservation [1,7,8]. A variety of radiation devices have been used to reach this goal. Proton and other charged particle therapies, together with episcleral radionuclide plaque therapy (brachytherapy), are some of the most commonly used [7-9]. Semenova et al. reported 10-year outcome data on 47 patients with T3 and T4 choroidal melanoma who underwent Pd-103 plaque radiation therapy. Enucleation was necessary in 11% of cases, while the local tumor control rate was 89% [8]. Macdonald and others reported an overall survival rate of 87.7% after proton therapy and an enucleation rate of 22.4% at a mean time of 23.8 months [10]. It is noteworthy that charged particle therapy requires a radiation treatment of several days with invasive fiducial implantation to deliver treatments safely [10].

In recent years, stereotactic radiosurgery (SRS) has become a well-established treatment. Gamma Knife-based SRS (Elekta AB, Stockholm, Sweden), as well as robotic SRS (CyberKnife), have the advantage of a single-session delivery treatment while not requiring invasive surgery for plaque implantation and removal with hospitalization for several days [3,5,11,12]. An essential requirement for accurate radiosurgery delivery is the immobilization of the ocular globe [7]. 

The Gamma Knife allows the use of both a stereotactic frame and a thermoplastic mask. Treatment is performed either under retrobulbar anesthesia or by suturing two to four rectus muscles. Others have described suction fixation devices [13]. Sarici et al. reported a tumor control rate of 90%, an eye retention rate of 82%, and an 18% incidence of metastasis [12]. A recent systematic review and meta-analysis about Gamma Knife radiosurgery for uveal melanoma reported a tumor control rate of 95% and a tumor regression rate of 83% [14].

Conversely to the Gamma Knife, robotic SRS does not require a rigid head fixation while achieving good results with standard retrobulbar anesthesia to achieve complete akinesia of the globe within the orbit without suturing the rectus muscles [2,15]. Eibl-Lindner et al. reported an actuarial three and five-year eye retention rate after robotic SRS of 86% and 73%, respectively. Local control at three and five years was 87.4% and 70.8%, respectively. The serviceable vision was maintained in 30.9% of patients at the last follow-up. The incidence of metastases was 11.5% [4]. Liegl et al. reported a local tumor control rate of 92.0% and 84.3% after three and five years for 21 to 22 Gy, and 86.9% and 77.7%, respectively, when the prescription dose was 20 Gy or less. Eye preservation was achieved in 89.9% and 81.0% after three and five years with 21 to 22 Gy and 85.9% and 80.0% for 20 Gy or less (70% isodose). Disease-specific survival rates were 93.1% after three years, 89.8% after five years, and 87.8% after seven years [3].

As with other SRS devices, GRS requires a rigorous setup with an experienced multidisciplinary team of imaging experts, neurosurgeons, radiation oncologists, and ophthalmologists. When used under optimal conditions, the described radiosurgical treatment is safe and comfortable for the patient. Further prospective clinical and dosimetric analyses are required for the final evaluation of GRS for uveal melanoma.

Radiosurgery workflows with retrobulbar anesthesia aim for a completion time of less than three hours to ensure akinesia of the globe until the end of treatment delivery [3,15]. With a total time to completion of approximately two hours, we have reported a successful adaptation of this treatment concept for GRS.

The characteristics of the GRS plan were mainly comparable to the robotic radiosurgery standard. Interestingly, the mean dose to the PTV of the GRS plan was higher by 10% while reducing the V10Gy to the surrounding healthy tissue at the same time. With treatment failure patterns in choroidal melanomas suggesting an advantage of higher doses to the tumor, this could potentially translate into improved local control without increased toxicity, although, according to a recent meta-analysis, treatment with a lower dose could be an option [2,14]. 

In the reported case, the entire procedure was well-tolerated by the patient, who was discharged from the outpatient clinic shortly after completion of the treatment. Further studies on the efficacy and safety of GRS for the treatment of uveal melanoma are necessary.

## Conclusions

This appears to be the first report of the use of GRS in uveal melanoma, to the best of our knowledge. The user and patient experience with the treatment platform was favorable, completing the radiosurgical procedure, including imaging studies, in less than three hours in an outpatient setting. The dose concepts and dosimetric parameters are mostly comparable to other SRS platforms.
